# Changes in quality of vitamin K antagonist treatment and clinical outcomes during Ramadan: a Dutch population-based cohort study

**DOI:** 10.1016/j.rpth.2025.103010

**Published:** 2025-08-13

**Authors:** Eva K. Kempers, Qingui Chen, Nienke van Rein, Ferdows Atiq, Roger K. Schindhelm, Nynke M. Wiersma, Charlotte E.A. Dronkers, Maarten Beinema, Arina J. ten Cate-Hoek, Melchior C. Nierman, Alexander D.M. Stork, Patricia Moriarty, Frederikus A. Klok, Suzanne C. Cannegieter, Marieke J.H.A. Kruip

**Affiliations:** 1 Department of Hematology, Erasmus MC, Erasmus University Medical Center, Rotterdam, the Netherlands; 2 Department of Clinical Epidemiology, Leiden University Medical Center, Leiden, the Netherlands; 3 Department of Clinical Pharmacy and Toxicology, Leiden University Medical Center, Leiden, the Netherlands; 4 Irish Centre for Vascular Biology, School of Pharmacy and Biomolecular Sciences, Royal College of Surgeons in Ireland, Dublin, Ireland; 5 Anticoagulation Clinic and Department of Clinical Chemistry, Diagnost-IQ, Hoorn, the Netherlands; 6 SaltroThrombosis Center Unilabs Midden, Department of Thrombosis and Anticoagulation, Utrecht, the Netherlands; 7 Department of Internal Medicine, Isala Hospital Zwolle, Zwolle, the Netherlands; 8 Anticoagulation Center, Deventer Hospital, Deventer, the Netherlands; 9 Thrombosis Expertise Center, Heart + Vascular Center, Maastricht University Medical Center (MUMC+), and Cardiovascular Research Institute Maastricht, Maastricht, the Netherlands; 10 Department of Thrombosis and Anticoagulation, Unilabs / Atalmedial Medical Diagnostic Centers, Amsterdam, the Netherlands; 11 Thrombosis Service Anna Hospital Geldrop, Geldrop, the Netherlands; 12 TromboseZorg Dichtbij, Druten, the Netherlands; 13 Department of Medicine - Thrombosis and Haemostasis, Leiden University Medical Center, Leiden, the Netherlands; 14 Department of Quality and Patient Care, Erasmus MC, Erasmus University Medical Center Rotterdam, Rotterdam, the Netherlands

**Keywords:** 4-hydroxycoumarins, hemorrhage, international normalized ratio, Ramadan fasting, thromboembolism

## Abstract

**Background:**

Ramadan fasting alters the timing and content of food, including dietary vitamin K, and medication intake, potentially affecting stability of vitamin K antagonist (VKA) treatment and clinical outcomes.

**Objectives:**

To assess population-level changes in VKA treatment quality and incidence of clinical events, including bleeding and venous and arterial thromboembolism, during Ramadan in the Netherlands.

**Methods:**

Data from 17 Dutch anticoagulation clinics were linked to Statistics Netherlands. Prevalent VKA users in 2013-2019 with an immigration background who were likely to fast during Ramadan (ie, the Ramadan cohort) were studied from 2 months preceding Ramadan until 2 months after. During each 30-day interval, VKA treatment quality and risk of clinical events were assessed. A cohort of native Dutch VKA users was studied as negative control.

**Results:**

The Ramadan cohort included 3835 VKA users (median age 65.8 years, 55.2% male). Frequency of international normalized ratio (INR) monitoring, INR variability, and time within target range remained similar across Ramadan. However, the proportion of supratherapeutic INRs was slightly higher during (18.9% ± 31.5% [mean ± SD]) and after Ramadan (month +1, 19.9% ± 32.7% and month +2, 19.7% ± 32.8%) compared with before (month –2, 18.1 ± 30.9% and month –1, 17.8 ± 31.5%). Meanwhile, there was a higher proportion of clinically relevant dose reductions during Ramadan (4.7%) than the other months (3.6%-4.3%). These were not observed in the native Dutch cohort (*N* = 139,207). Monthly risk of the composite of bleeding and thromboembolic events remained unchanged across Ramadan in both cohorts.

**Conclusion:**

There were no clinically relevant population-level changes across Ramadan in VKA treatment quality and clinical outcomes, except for a slightly higher proportion of supratherapeutic INRs and dose reductions.

## Essentials

• Ramadan-related changes in diet and medication intake may impact quality of vitamin K antagonist (VKA) therapy.
• This cohort study assessed VKA treatment quality and clinical outcomes across Ramadan at a population level.
• Supratherapeutic anticoagulation intensity and VKA dose reductions occurred slightly more often during Ramadan.
• Rates of death, bleeding, and thromboembolic events remained unchanged during the Ramadan period.

## 1 Introduction

Although direct oral anticoagulants (DOACs) have replaced vitamin K antagonists (VKAs) as first-choice oral anticoagulant treatment for most common indications, including stroke prevention in atrial fibrillation (AF) and treatment or prevention of venous thromboembolism [[1], [2], [3]], VKAs remain indicated in patients with mechanical heart valves [4,5], (rheumatic) moderate-to-severe mitral stenosis [6], or antiphospholipid syndrome [[7], [8], [9], [10], [11]]. In addition, among those who were already receiving VKAs, switching to DOACs may not always be appropriate; for instance, in frail older AF patients, this switch was associated with increased bleeding risk [12]. Globally, VKAs are still commonly used, partly because of cost-related issues for DOACs [13,14]. Treatment with VKAs has some disadvantages, such as the narrow therapeutic window and multiple drug and food interactions [[15], [16], [17], [18], [19], [20]]. Changes in dietary intake of vitamin K affect their anticoagulant effect [[21], [22], [23], [24], [25], [26], [27]]. Therefore, patients treated with VKAs are usually managed by specialized anticoagulation clinics, which monitor the international normalized ratio (INR) and perform dose adjustments when necessary [16]. In addition, VKA users are advised to maintain a stable dietary vitamin K intake [16].

During Ramadan, the ninth month of the Islamic lunar calendar, adult Muslims are expected to fast, which includes refraining from eating and drinking, including oral medication intake, from dawn to sunset [28]. The month of Ramadan typically lasts for 29 to 30 days. Muslims are exempted when fasting may cause significant harm to their health [[29], [30], [31], [32]]. Changes in dietary vitamin K intake during Ramadan and timing of food and oral medication intake, which is limited to the predawn and meal at sunset [28,33], may cause fluctuations in stability of VKA treatment. Muslims on long-term anticoagulant treatment with warfarin or DOACs who fasted during Ramadan reported that self-guided modifications of oral anticoagulant intake occur frequently during Ramadan, including adjusted timing of intake and skipping or doubling doses, to adapt to the fasting times [34].

A limited number of studies have shown inconsistent results on the potential impact of Ramadan fasting on quality of VKA treatment and clinical outcomes [[35], [36], [37], [38], [39], [40], [41], [42], [43]]. A systematic review of studies comparing cohorts of patients before, during, and after Ramadan reported no significant change in time in therapeutic range (TTR) but a slightly higher mean INR during Ramadan and more frequent supratherapeutic INRs during and after it [43]. Nevertheless, these studies were limited by their sample sizes, and not all assessed the occurrence of clinical outcomes.

By identifying a cohort of VKA users who are likely to fast during Ramadan, we intend to overcome these limitations and assess potential population-level changes in VKA treatment quality and incidences of adverse clinical events, including bleeding and venous and arterial thromboembolic events, across Ramadan in the Netherlands.

## 2 Methods

### 2.1 Setting and data sources

In the Netherlands, patients treated with VKAs are managed by regional anticoagulation clinics [44]. Seventeen Dutch anticoagulation clinics provided detailed data on VKA treatment, including start and end dates of VKA therapy, treatment indications, VKA type and dose, INR target range, and INR results [45]. Data from these clinics were linked on an individual level to nationwide data from Statistics Netherlands (in Dutch, “Centraal Bureau voor de Statistiek” [CBS]) by sex, date of birth, postal code, and last known date to be alive. CBS provides nationwide data on personal characteristics [46], diagnoses made during hospital admissions in Dutch hospitals [[47], [48], [49], [50]], outpatient-dispensed medication prescriptions [51], cause of death [52], and date of death [53]. Diagnoses in the Dutch Hospital Data registry are coded according to the *International Classification of Diseases* (ICD) (ICD-9 for some diagnoses made from 2010 to 2012, and ICD-10 thereafter). Data on outpatient-dispensed medication prescriptions include year of dispensing and Anatomical Therapeutic Chemical code (4 digits) and for anticoagulants only, dispensing dates and anticoagulant type. More information on the data sources used is described in the Supplementary Methods.

### 2.2 Study population

Our primary study cohort, ie, the Ramadan cohort, comprised prevalent VKA users, treated at one of the participating anticoagulation clinics between 2013 and 2019, who were likely to fast during Ramadan. VKA users were classified as such based on an immigration background (first or second generation) from a country of origin where the Muslim population accounts for ≥85% of the total population (Supplementary Methods and Supplementary Table 1). During the study period, approximately 5% of the total population in the Netherlands identified as Muslim, with the largest groups originating from Turkey or Morocco [54,55]. We included adults (aged ≥18 years) who were already receiving VKA treatment ≥2 months before the start of Ramadan (d_0_) in a particular calendar year from 2013 to 2019. Exclusion criteria were 1) no registered dispensed VKA prescription or a registered dispensed DOAC prescription within 6 months before the start of follow-up (d_0_), 2) a type of VKA other than the 2 registered VKAs in the Netherlands (acenocoumarol and phenprocoumon), 3) missing INR target range at the last INR measurement before d_0_, or 4) country of origin imputed by CBS.

As a negative control, we similarly identified a cohort of VKA users with the Netherlands as their registered country of origin (ie, native Dutch cohort), applying the same inclusion and exclusion criteria. For both cohorts, if a VKA user was eligible for inclusion over multiple calendar years, only 1 calendar year was randomly selected for inclusion.

### 2.3 Study design

**Figure 1:**
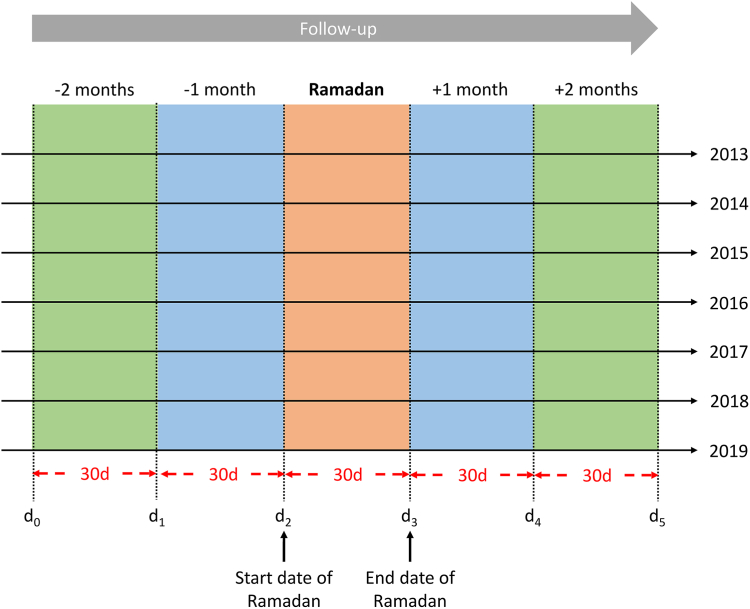
Study design. For each calendar year during the study period (2013-2019), follow-up started 2 months before the start of Ramadan (d_0_) and ended 2 months after the end of Ramadan (d_5_). The 30-day intervals were determined based on the start and end dates of Ramadan in a particular calendar year. Note: d_2_ and d_3_ refer to the start date and end date of Ramadan in each calendar year, respectively, but to keep the interval at 30 days consistently, d_3_ of years 2015-2019 was the actual end date of Ramadan plus 1 more day.

For each calendar year (2013-2019), follow-up started 2 months before the start of Ramadan (d_0_) and ended 2 months after the end of Ramadan (d_5_) (Figure 1). Thirty-day intervals were determined based on the start and end dates of Ramadan in a particular calendar year. Potential changes in quality of VKA treatment and the incidence of adverse clinical events across Ramadan were assessed by comparing these metrics across the 5 30-day observation periods within the same cohort (either the Ramadan cohort or the native Dutch cohort).

### 2.4 Baseline characteristics

Baseline characteristics were collected at d_0_ and included age, sex, immigration background (ie, native Dutch, first or second-generation immigrant), INR target range, type of INR monitoring (eg, at home, outpatient, self-management), type of VKA (acenocoumarol or phenprocoumon), presence of comorbidities, CHA_2_DS_2_-VASc and HAS-BLED scores, and prior use of low molecular weight heparin or antiplatelet drugs (defined as ≥1 dispensed prescription within 6 months before d_0_). Comorbidities were identified by hospital diagnoses registered within 3 years before d_0_ (Supplementary Table 2).

### 2.5 Study outcomes

The quality of VKA treatment was assessed within each 30-day period, with 1 period corresponding to Ramadan. We studied the following metrics: median INR value, median recommended average dose, ≥1 clinically relevant dose increases or reductions, proportions of INRs within/below/above the target range, proportions of INRs ≥5 or ≥8, percentage of time in/below/above the target range (TTR, TBR, and TAR, respectively), and INR variability. A clinically relevant dose adjustment was defined as a change of ≥10% in the recommended average VKA dose prescribed at 2 consecutive INR measurements. TTR, TBR, and TAR were calculated according to the Rosendaal method, which assumes a linear relationship between consecutive INR measurements and divides the time with linearly interpolated INR values within, below or above the INR target range by the total number of observation days [56]. INR variability was expressed by the variance growth rate (VGR) according to Cannegieter, which reflects the deviation in INR value between 2 consecutive measurements [57]. Only individuals receiving VKA treatment at the start of a 30-day observation period and with ≥2 INR measurements within a certain 30-day period (where 1 INR measurement was allowed to be recorded in the previous observation period) were included in this calculation. In addition, we evaluated the frequency of INR monitoring, the time interval between 2 consecutive INR measurements, and switch to another type of VKA (according to anticoagulation clinic records) or DOAC (according to outpatient-dispensed prescriptions).

Within each 30-day observation period, we determined if adverse clinical events occurred, including 1) major/clinically relevant bleeding, 2) arterial thromboembolism (ischemic stroke, transient ischemic attack [TIA], myocardial infarction, and other arterial thromboembolism), 3) the composite of venous and arterial thromboembolic and bleeding events, and 4) all-cause mortality. These events were identified by first in-hospital diagnoses (restricted to primary or main diagnosis) and primary causes of death (Supplementary Table 2).

Individuals were followed from d_0_ until 2 months after the end of Ramadan or until a registered DOAC dispensing, registered DOAC use according to anticoagulation clinic records, discontinuation of VKA therapy, a clinical event of interest, or date of death, whichever occurred first.

### 2.6 Statistical analyses

Summary statistics of the baseline characteristics as well as the metrics for the quality of VKA treatment are presented as mean ± SD, median (25th-75th percentile, IQR), or frequency (percentage). To assess differences in the continuous metrics for the quality of VKA treatment across Ramadan within the same cohort, a linear mixed-effects model was employed. The 5 observation periods were treated as a 5-level categorical variable with the first period as reference (ie, month −2 prior to Ramadan), and a subject-specific random intercept was included to account for within-subject correlations over time. Model parameters were estimated via restricted maximum likelihood, with 95% CIs derived by the Wald method.

For clinical outcomes, 30-day cumulative incidences were estimated by the cumulative incidence competing risks method or Kaplan–Meier method (for all-cause mortality) within each of the 5 observations periods (ie, month −2 prior to Ramadan, month −1 prior to Ramadan, Ramadan month, month +1 after Ramadan, month +2 after Ramadan). Incidence rates were calculated by dividing the number of first events by total observation time, with 95% CIs according to the chi-squared distribution.

We performed a subgroup analysis among VKA users in the Ramadan cohort with Morocco as their registered country of origin. This was supported by a survey study from The Netherlands Institute for Social Research indicating that the vast majority of Dutch Moroccan Muslims fasted every day during Ramadan (87%), while lower participation rates were reported for Muslims with other backgrounds [58]. In addition, participation was higher among first-generation than second-generation immigrants.

Analyses were performed in R (version 4.4.0, R Core Team) [59] with the packages *dplyr* [60], *tableone* [61], *survival* [62], *cmprsk* [63], and *lme4* [64].

### 2.7 Ethics approval

This study used deidentified, retrospective administrative data from population registries and routine care in the Netherlands. Consequently, it was exempt from ethics approval under the Dutch Medical Research Involving Human Subjects Act (WMO), and individual participant consent was waived. The Scientific Committee of the Department of Clinical Epidemiology of the Leiden University Medical Centre approved the study protocol (No. A232). Results based on a frequency <10 were masked in line with CBS’s privacy policy.

## 3 Results

### 3.1 Study population

**Figure 2:**
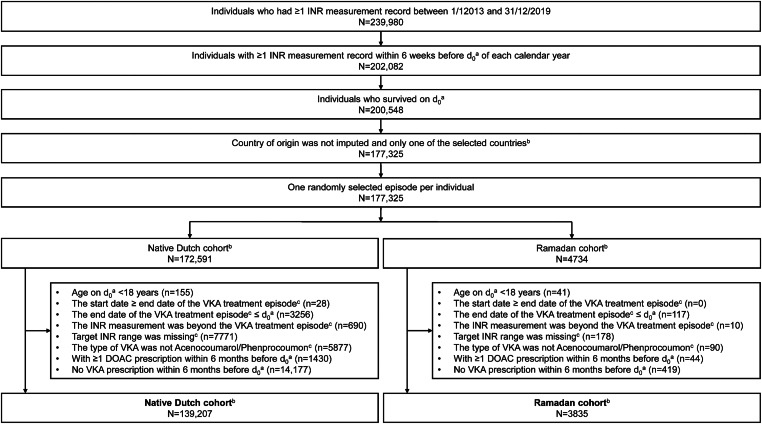
Flowchart of the Ramadan cohort and Native Dutch cohort. ^a^The date d_0_ refers to the date 60 days before the start of Ramadan in a calendar year. ^b^Details about the identification of the Ramadan cohort are presented in the Supplementary Methods. ^c^Based on data on the latest INR measurement record within 6 weeks before d_0_. In the data, the other registered types of anticoagulants included warfarin, fluindione, direct oral anticoagulant, or unknown. DOAC, direct oral anticoagulant; INR, international normalized ratio; VKA, vitamin K antagonist.

Among 239,980 VKA users between 2013 and 2019, a total of 4734 VKA users were considered eligible for inclusion in the Ramadan cohort as well as 172,591 for the native Dutch cohort. After applying the exclusion criteria, a total of 3835 VKA users were included in the Ramadan cohort and 139,207 in the native Dutch cohort (Figure 2).

The majority of VKA users in the Ramadan cohort were of Moroccan (38.9%) or Turkish (34.4%) origin and were first-generation immigrants (95.9%) (Table 1). The Ramadan cohort had a median age of 65.8 (IQR, 55.0-74.9) years, and 55.2% of VKA users were male. Most patients were managed with INR target ranges of 2.0 to 3.0 or 2.5 to 3.5 (89.0%) and used acenocoumarol (82.1%). The most commonly registered indications for VKA treatment were AF (56.3%) and other valvular heart disease (18.2%), followed by venous thromboembolism (15.5%). The median CHA_2_DS_2_-VASc and HAS-BLED scores were 2 (IQR, 1-3) and 1 (IQR, 1-2), respectively. Based on in-hospital diagnoses within 3 years before d_0_, 3.3% had a history of ischemic stroke/TIA and 6.2% of major bleeding (Supplementary Table 3).

**Table 1:** Baseline characteristics of study cohorts.

Cohort	Ramadan cohort (*N* = 3835)	Native Dutch cohort (*N* = 139,207)
Age (y), median (IQR)	65.8 (55.0-74.9)	76.7 (68.4-84.0)
Female, *n* (%)	1717 (44.8)	62,764 (45.1)
Immigration background, *n* (%)		
Native Dutch	0 (0)	139,207 (100)
First-generation immigrant	3678 (95.9)	0 (0)
Second-generation immigrant	157 (4.1**)**	0 (0)
Country of origin, *n* (%)		
Netherlands	0 (0)	139,207 (100)
Morocco	1492 (38.9)	0 (0)
Turkey	1321 (34.4)	0 (0)
Indonesia	272 (7.1)	0 (0)
Iraq	143 (3.7)	0 (0)
Other	607 (15.8)	0 (0)
Calendar year of d_0_^a^, *n* (%)		
2013	406 (10.6)	18,527 (13.3)
2014	503 (13.1)	18,771 (13.5)
2015	567 (14.8)	18,728 (13.5)
2016	579 (15.1)	19,733 (14.2)
2017	551 (14.4)	20,669 (14.8)
2018	541 (14.1)	18,244 (13.1)
2019	688 (17.9)	24,535 (17.6)
INR target range, *n* (%)		
2.0-3.0	1637 (42.7)	71,580 (51.4)
2.5-3.5	1776 (46.3)	58,622 (42.1)
3.0-4.0	394 (10.3)	7876 (5.7)
Other	28 (0.7)	1129 (0.8)
Type of VKA, *n* (%)		
Acenocoumarol	3149 (82.1)	107,178 (77.0)
Phenprocoumon	686 (17.9)	32,029 (23.0)
Type of INR monitoring, *n* (%)		
At home measurement	1447 (37.7)	50,983 (36.6)
Nursing home	<10^b^	2020 (1.5)
Outpatient	1993 (52.0)	65,756 (47.2)
Self-management	57 (1.5)	5859 (4.2)
Self-measurement	306 (8.0)	12,481 (9.0)
Unknown	Masked^b^	2108 (1.5)
Registered VKA treatment indication^c^, *n* (%)		
Atrial fibrillation	2158 (56.3)	98,640 (70.9)
Stroke/transient ischemic attack	109 (2.8)	4781 (3.4)
Systemic thromboembolism/peripheral arterial disease	90 (2.3)	2826 (2.0)
Heart failure	168 (4.4)	2787 (2.0)
Venous thromboembolism	596 (15.5)	21,768 (15.6)
Mechanical heart valve	537 (14.0)	7305 (5.2)
Biovalve prosthesis	75 (2.0)	2825 (2.0)
Rheumatic mitral stenosis	<10^b^	139 (0.1)
Other mitral valve disease	53 (1.4)	980 (0.7)
Aortic valve disease	26 (0.7)	1058 (0.8)
Other valve disease	697 (18.2)	10,090 (7.2)
Coronary artery disease	96 (2.5)	3548 (2.5)
Cardiomyopathy	269 (7.0)	4827 (3.5)
Antiphospholipid syndrome/thrombophilia	<10^b^	92 (0.1)
Other	338 (8.8)	12,440 (8.9)
CHA_2_DS_2_-VASc score, median (IQR)	2 (1-3)	2 (1-3)
HAS-BLED score^d^, median (IQR)	1 (1-2)	1 (1-2)

INR, international normalized ratio; VKA, vitamin K antagonist.

a The date d_0_ refers to the date 60 days before the start of Ramadan in a particular calendar year.

b Cells containing <10 individuals are masked according to Statistics Netherlands’ privacy policy. To prevent recalculation of cells <10, sometimes corresponding cells are additionally masked.

c All indications for VKA treatment that have been registered in the data are presented, regardless of whether an indication became valid before d_0_ (because of data availability). One or more indications can be present.

d Calculated without the term “labile INR.”

Compared with the Ramadan cohort, the native Dutch cohort was older (median age 76.7 [IQR, 68.4-84.0] years) and had a different distribution of indications for VKA treatment (ie, 70.9% AF, 7.2% other valvular heart disease; Table 1). Additional baseline characteristics of the study cohorts, such as relevant comorbidities and prior use of antithrombotic drugs, are displayed in Supplementary Tables 3 and 4.

### 3.2 Quality of VKA treatment across Ramadan

**Figure 3:**
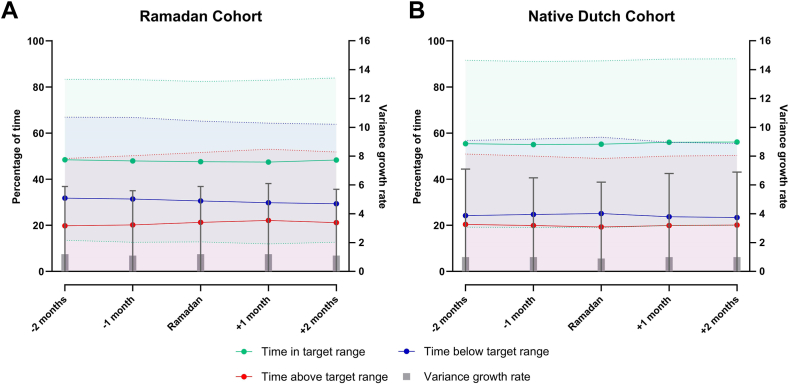
Quality of vitamin K antagonist treatment across Ramadan. Quality of vitamin K antagonist treatment for each 30-day period during follow-up is expressed as percentage of time in, above, and below target range (TTR, TAR, and TBR) and by the variance growth rate, which is a measure for international normalized ratio variability. TTR, TAR, and TBR were calculated according to the Rosendaal method. For all metrics, mean and SD are displayed separately for the (A) Ramadan and (B) native Dutch cohorts.

In the Ramadan cohort, TTR remained low but stable across Ramadan (Figure 3A), with a mean TTR during Ramadan of 47.6 ± 34.8 and the lowest TTR during the month after Ramadan (47.4 ± 35.5) (Table 2). The mean proportion of INRs within target range was 51.2% ± 41.0% during Ramadan, which was slightly lower compared with the other months (ranging from 51.5% to 53.0%), although not different than the first observation period (ie, month −2 prior to Ramadan; difference of −0.4% [95% CI, −2.3% to 1.5%]). Both during and after Ramadan (Ramadan month, month +1, and month +2), a higher mean proportion of INRs was above target range (18.9% ± 31.5%, 19.9% ± 32.7%, 19.7% ± 32.8%, respectively) compared with the months preceding Ramadan (month −2, 18.1% ± 30.9% and month −1, 17.8% ± 31.5%). Compared to the first observation period, the mean proportions of INRs above target were on average 0.8%, 1.8%, and 1.6% higher, respectively, during Ramadan and the next 2 months. INR variability remained similar across Ramadan, with a mean VGR of 1.2 ± 4.7 during Ramadan.

**Table 2:** Vitamin K antagonist treatment across Ramadan.

Cohort	Ramadan cohort					Native Dutch cohort				
Observation period	−2 month	−1 month	Ramadan	+1 month	+2 month	-2 month	-1 month	Ramadan	+1 month	+2 month
Number of INR measurements	3835^d^	3718^d^	3633^d^	3531^d^	3459^d^	139,207^d^	135,402^d^	132,036^d^	128,779^d^	125,851^d^
Median (IQR)	2.0 (1.0-3.0)	1.0 (1.0-2.0)	1.0 (1.0-2.0)	1.0 (1.0-2.0)	1.0 (1.0-2.0)	1.0 (1.0-2.0)	1.0 (1.0-2.0)	1.0 (1.0-2.0)	1.0 (1.0-2.0)	1.0 (1.0-2.0)
Mean interval between INR measurement (d)	3422^d^	3051^d^	2864^d^	2674^d^	2509^d^	127,345^d^	115,712^d^	112,304^d^	109,611^d^	106,174^d^
Median (IQR)	16.3 (10.5-24.5)	16.0 (10.8-22.0)	16.3 (10.5-22.5)	16.3 (11.0-23.0)	17.0 (11.3-23.5)	18.0 (12.0-28.0)	17.5 (12.0-28.0)	17.5 (13.0-28.0)	18.7 (13.7-28.0)	19.0 (13.5-28.0)
Median INR value	3427^d^	3212^d^	3009^d^	2811^d^	2684^d^	127,410^d^	122,520^d^	119,490^d^	116,501^d^	112,972^d^
Median (IQR)	2.60 (2.20-3.10)	2.60 (2.20-3.10)	2.60 (2.20-3.10)	2.60 (2.20-3.20)	2.65 (2.20-3.20)	2.70 (2.30-3.10)	2.60 (2.30-3.10)	2.60 (2.30-3.05)	2.65 (2.30-3.10)	2.65 (2.30-3.10)
Mean ± SD	2.71 ± 0.79	2.70 ± 0.74	2.71 ± 0.78	2.74 ± 0.79	2.74 ± 0.77	2.74 ± 0.68	2.72 ± 0.67	2.70 ± 0.66	2.73 ± 0.68	2.73 ± 0.68
Fixed effect β (95% CI)	0 (Reference)	−0.017 (−0.050 to 0.016)	−0.004 (−0.038 to 0.030)	0.021 (−0.014 to 0.056)	0.013 (−0.023 to 0.048)	0 (Reference)	−0.019 (−0.024 to −0.015)	−0.035 (−0.040 to −0.030)	−0.011 (−0.015 to −0.006)	−0.005 (−0.010 to 0.000)
Median recommended average dose^a^	3423^d^	3208^d^	3007^d^	2807^d^	2680^d^	127,300^d^	122,413^d^	119,390^d^	116,400^d^	112,881^d^
Median (IQR)	2.00 (1.29-2.91)	2.00 (1.29-2.89)	2.00 (1.28-2.90)	2.00 (1.27-2.91)	1.94 (1.27-2.86)	1.78 (1.00-2.57)	1.77 (1.01-2.57)	1.75 (1.00-2.57)	1.75 (1.00-2.57)	1.75 (1.00-2.57)
Mean ± SD	2.21 ± 1.30	2.22 ± 1.31	2.19 ± 1.29	2.20 ± 1.29	2.18 ± 1.28	1.93 ± 1.16	1.93 ± 1.16	1.92 ± 1.15	1.91 ± 1.15	1.90 ± 1.14
Fixed effect β (95% CI)	0 (Reference)	0.001 (−0.008 to 0.011)	−0.012 (−0.021 to −0.002)	−0.021 (−0.031 to −0.011)	−0.024 (−0.034 to −0.014)	0 (Reference)	−0.006 (−0.007 to −0.005)	−0.009 (−0.011 to −0.008)	−0.015 (−0.017 to −0.014)	−0.022 (−0.024 to −0.021)
≥1 clinically relevant dose increase^b^	3417^d^	3045^d^	2855^d^	2666^d^	2501^d^	127,143^d^	115,440^d^	112,066^d^	109,384^d^	105,952^d^
*n* (%)	126 (3.7)	94 (3.1)	91 (3.2)	73 (2.7)	72 (2.9)	4911 (3.9)	4132 (3.6)	3868 (3.5)	3430 (3.1)	3182 (3.0)
≥1 clinically relevant dose reduction^b^	3417^d^	3045^d^	2855^d^	2666^d^	2501^d^	127,143^d^	115,440^d^	112,066^d^	109,384^d^	105,952^d^
*n* (%)	146 (4.3)	112 (3.7)	135 (4.7)	107 (4.0)	90 (3.6)	5766 (4.5)	4811 (4.2)	4485 (4.0)	4378 (4.0)	4137 (3.9)
INR variability (VGR)	3422^d^	3051^d^	2864^d^	2674^d^	2509^d^	127,345^d^	115,712^d^	112,304^d^	109,611^d^	106,174^d^
Mean ± SD	1.2 ± 4.7	1.1 ± 4.5	1.2 ± 4.7	1.2 ± 4.9	1.1 ± 4.6	1.0 ± 6.1	1.0 ± 5.5	0.9 ± 5.3	1.0 ± 5.8	1.0 ± 5.9
Fixed effect β (95% CI)	0 (Reference)	−0.124 (−0.339 to 0.091)	−0.031 (−0.250 to 0.189)	0.070 (−0.154 to 0.294)	−0.106 (−0.334 to 0.122)	0 (Reference)	−0.056 (−0.099 to −0.013)	−0.117 (−0.160 to −0.073)	−0.038 (−0.082 to 0.005)	−0.012 (−0.056 to 0.032)
Proportion of INRs within target range, %	3427^d^	3212^d^	3009^d^	2811^d^	2684^d^	127,410^d^	122,520^d^	119,490^d^	116,501^d^	112,972^d^
Mean ± SD	51.5 ± 40.9	53.0 ± 41.9	51.2 ± 41.0	51.9 ± 41.8	51.9 ± 42.0	59.4 ± 41.1	60.0 ± 41.5	60.3 ± 41.4	60.9 ± 41.3	60.9 ± 41.4
Fixed effect β (95% CI)	0 (Reference)	1.449 (−0.434 to 3.332)	−0.419 (−2.337 to 1.500)	0.295 (−1.662 to 2.252)	0.289 (−1.695 to 2.274)	0 (Reference)	0.741 (0.438-1.043)	1.098 (0.793-1.402)	1.611 (1.305-1.918)	1.642 (1.333-1.952)
Proportion of INRs below target range, %	3427^d^	3212^d^	3009^d^	2811^d^	2684^d^	127,410^d^	122,520^d^	119,490^d^	116,501^d^	112,972^d^
Mean ± SD	30.4 ± 37.4	29.2 ± 38.0	29.9 ± 37.7	28.2 ± 37.4	28.4 ± 37.6	22.3 ± 34.4	22.4 ± 35.0	22.8 ± 35.3	21.6 ± 34.3	21.3 ± 34.3
Fixed effect β (95% CI)	0 (Reference)	−1.085 (−2.738 to 0.568)	−0.308 (−1.993 to 1.377)	−1.951 (−3.671 to −0.231)	−1.764 (−3.509 to −0.020)	0 (Reference)	0.038 (−0.206 to 0.283)	0.426 (0.180-0.672)	−0.766 (−1.014 to −0.519)	−0.990 (−1.240 to −0.740)
Proportion of INRs above target range, %	3427^d^	3212^d^	3009^d^	2811^d^	2684^d^	127,410^d^	122,520^d^	119,490^d^	116,501^d^	112,972^d^
Mean ± SD	18.1 ± 30.9	17.8 ± 31.5	18.9 ± 31.5	19.9 ± 32.7	19.7 ± 32.8	18.3 ± 31.8	17.6 ± 31.7	16.9 ± 31.0	17.6 ± 31.7	17.8 ± 31.8
Fixed effect β (95% CI)	0 (Reference)	−0.307 (−1.787 to 1.173)	0.802 (−0.705 to 2.310)	1.783 (0.247-3.320)	1.586 (0.028-3.144)	0 (Reference)	−0.772 (−1.008 to −0.536)	−1.512 (−1.750 to −1.275)	−0.823 (−1.062 to −0.584)	−0.623 (−0.864 to −0.382)
Proportion of INRs ≥5, %	3427^d^	3212^d^	3009^d^	2811^d^	2684^d^	127,410^d^	122,522^d^	119,493^d^	116,504^d^	112,978^d^
Mean ± SD	2.7 ± 11.7	2.3 ± 10.6	2.7 ± 11.8	3.0 ± 12.2	2.6 ± 11.7	1.9 ± 9.5	1.8 ± 9.2	1.7 ± 9.0	1.8 ± 9.3	1.8 ± 9.3
Proportion of INRs ≥8, %	3427^d^	3212^d^	3009^d^	2811^d^	2684^d^	127,410^d^	122,522^d^	119,493^d^	116,504^d^	112,978^d^
Mean ± SD	0.3 ± 3.4	0.2 ± 2.7	0.2 ± 3.1	0.2 ± 3.4	0.1 ± 2.1	0.3 ± 3.3	0.2 ± 3.0	0.2 ± 3.0	0.2 ± 3.0	0.2 ± 3.2
Time in target range, %	3400^d^	3041^d^	2852^d^	2653^d^	2496^d^	126,350^d^	115,261^d^	111,666^d^	109,051^d^	105,748^d^
Mean ± SD	48.4 ± 34.9	47.9 ± 35.3	47.6 ± 34.8	47.4 ± 35.5	48.3 ± 35.6	55.4 ± 36.2	55.0 ± 36.0	55.2 ± 36.2	56.0 ± 36.1	56.1 ± 36.1
Fixed effect β (95% CI)	0 (Reference)	−0.370 (−1.958 to 1.217)	−0.669 (−2.292 to 0.954)	−0.977 (−2.635 to 0.681)	0.065 (−1.624 to 1.755)	0 (Reference)	−0.068 (−0.332 to 0.195)	0.418 (0.151-0.685)	1.076 (0.808-1.345)	1.210 (0.939-1.481)
Time below target range, %	3400^d^	3041^d^	2852^d^	2653^d^	2496^d^	126,350^d^	115,261^d^	111,666^d^	109,051^d^	105,748^d^
Mean ± SD	31.8 ± 35.1	31.4 ± 35.4	30.6 ± 34.6	29.8 ± 34.5	29.4 ± 34.4	24.2 ± 32.6	24.7 ± 32.7	25.1 ± 33.1	23.7 ± 32.3	23.4 ± 32.1
Fixed effect β (95%CI)	0 (Reference)	−0.404 (−1.943 to 1.135)	−1.245 (−2.820 to 0.330)	−1.842 (−3.451 to −0.232)	−2.264 (−3.904 to −0.624)	0 (Reference)	0.326 (0.096-0.555)	0.564 (0.331-0.797)	−0.728 (−0.962 to −0.494)	−1.000 (−1.236 to −0.764)
Time above target range, %	3400^d^	3041^d^	2852^d^	2653^d^	2496^d^	126,350^d^	115,261^d^	111,666^d^	109,051^d^	105,748^d^
Mean ± SD	19.8 ± 29.2	20.2 ± 30.0	21.3 ± 30.3	22.1 ± 30.9	21.2 ± 30.6	20.4 ± 30.5	19.9 ± 30.1	19.3 ± 29.7	19.9 ± 30.1	20.1 ± 30.2
Fixed effect β (95% CI)	0 (Reference)	0.364 (−1.039 to 1.768)	1.423 (−0.010 to 2.855)	2.202 (0.739-3.665)	1.259 (−0.231 to 2.749)	0 (Reference)	−0.613 (−0.838 to −0.388)	−1.360 (−1.588 to −1.132)	−0.695 (−0.924 to −0.465)	−0.534 (−0.766 to −0.303)
Changed VKA type	3416^d^	3041^d^	2851^d^	2665^d^	2496^d^	127,096^d^	115,343^d^	111,978^d^	109,265^d^	105,835^d^
*n* (%)	<10^c^	<10^c^	<10^c^	<10^c^	<10^c^	120 (0.1)	141 (0.1)	97 (0.1)	95 (0.1)	73 (0.1)
Switch to DOACs	3835^d^	3718^d^	3633^d^	3531^d^	3459^d^	139,207^d^	135,402^d^	132,036^d^	128,779^d^	125,851^d^
*n* (%)	18 (0.5)	12 (0.3)	12 (0.3)	Masked^c^	<10^c^	438 (0.3)	352 (0.3)	337 (0.3)	306 (0.2)	258 (0.2)

DOAC, direct oral anticoagulant; INR, international normalized ratio; VGR, variance growth rate; VKA, vitamin K antagonist.

a Recommended average dose is expressed as number of tablets, where 1 tablet of acenocoumarol contains 1 mg and 1 tablet of phenprocoumon contains 3 mg.

b A clinically relevant dose increase or reduction was defined as a change of ≥10% in the average daily VKA dose prescribed at 2 consecutive INR measurements.

c Cells containing <10 individuals were masked according to Statistics Netherlands’ privacy policy. To prevent recalculation of cells <10, sometimes corresponding cells are additionally masked.

d Number of individuals included in calculation for particular 30-day period.

Meanwhile, there was a higher proportion of clinically relevant dose reductions during Ramadan (4.7%) than the months before (3.7%-4.3%) and after Ramadan (3.6%-4.0%). In contrast, the proportion of clinically relevant dose increases was lower during the months after Ramadan (2.7%-2.9%) than the pre- and Ramadan period (3.1%-3.7%). However, the median recommended average VKA dose did not differ across Ramadan. The frequency of INR monitoring was also similar across the different periods with a median of 1 measurement per month (IQR, 1-2) and a median of approximately 16 days between consecutive INR measurements.

In comparison, VKA treatment quality metrics in the negative control native Dutch cohort remained similar across Ramadan, including time and percentage of INRs within target range, and VGR (Figure 3B and Table 2). The mean TTR during the Ramadan month was 55.2 ± 36.2 and on average 60.3% ± 41.4% of INRs were within target range.

### 3.3 Clinical events across Ramadan

**Figure 4:**
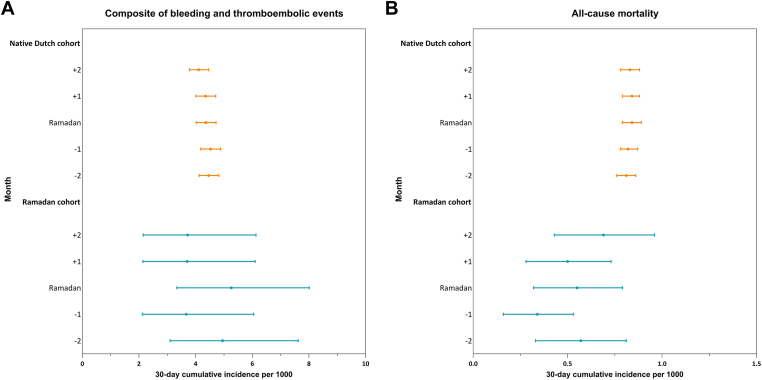
30-day cumulative incidence of clinical outcomes across Ramadan. 30-day cumulative incidences for both (A) the composite of bleeding and thromboembolic events and (B) all-cause mortality with corresponding 95% CIs are depicted separately for the native Dutch (orange) and Ramadan (blue) cohorts. The 5 observation periods are displayed as: month −2 prior to Ramadan, month −1 prior to Ramadan, Ramadan month, month +1 after Ramadan, and month +2 after Ramadan. Thromboembolic events included venous thromboembolism, ischemic stroke, transient ischemic attack, myocardial infarction, and other arterial thromboembolism.

A low number of bleeding events was observed among the Ramadan cohort during the 5-month study period. The 30-day cumulative incidence of major and clinically relevant bleeding during Ramadan was 2.63 (95% CI, 1.37-4.74) per 1000 (Table 3). For the composite outcome of bleeding, venous and arterial thromboembolic events, the 30-day cumulative incidence per 1000 was numerically the highest during Ramadan, at 5.26 (95% CI, 3.34-8.01), compared to the 2 preceding months (4.95; 95% CI, 3.10-7.62 and 3.67; 95% CI, 2.12-6.05, respectively) or the 2 months after Ramadan (3.70; 95% CI, 2.14-6.10 and 3.72; 95% CI, 2.15-6.13, respectively), although with overlapping CIs (Figure 4A). A consistent pattern was observed when evaluated by incidence rates (Table 3). Cumulative 30-day all-cause mortality was similar across the 5 observation periods, ranging from 0.34 to 0.69 per 1000.

**Table 3:** Incidence of clinical outcomes across Ramadan.

Cohort	Ramadan cohort					Native Dutch cohort				
Observation period	−2 month	−1 month	Ramadan	+1 month	+2 month	-2 month	-1 month	Ramadan	+1 month	+2 month
No. at risk	3835	3813	3800	3779	3760	139,207	138,079	136,940	135,785	134,651
Major/clinically relevant bleeding										
No. of events	<10^a^	<10^a^	10	<10^a^	<10^a^	273	266	235	264	235
IR (95% CI), per 100 PYs	Masked^a^	Masked^a^	3.22 (1.54-5.92)	Masked^a^	Masked^a^	2.40 (2.12-2.70)	2.36 (2.08-2.66)	2.10 (1.84-2.39)	2.38 (2.10-2.68)	2.13 (1.87-2.43)
30-d cumulative incidence, per 1000 (95% CI)	Masked^a^	Masked^a^	2.63 (1.37-4.74)	Masked^a^	Masked^a^	1.96 (1.74-2.21)	1.93 (1.71-2.17)	1.72 (1.51-1.95)	1.94 (1.72-2.19)	1.75 (1.53-1.98)
Ischemic stroke/TIA/myocardial infarction/arterial thromboembolism										
No. of events	12	11	<10^a^	<10^a^	12	324	337	338	299	284
IR (95% CI), per 100 PYs	3.82 (1.98-6.68)	3.52 (1.76-6.31)	Masked^a^	Masked^a^	3.90 (2.02-6.82)	2.85 (2.55-3.17)	2.99 (2.68-3.32)	3.02 (2.71-3.36)	2.69 (2.40-3.02)	2.58 (2.29-2.90)
30-d cumulative incidence, per 1000 (95% CI)	3.13 (1.73-5.36)	2.88 (1.55-5.06)	Masked^a^	Masked^a^	3.19 (1.76-5.47)	2.33 (2.09-2.59)	2.44 (2.19-2.71)	2.47 (2.22-2.74)	2.20 (1.96-2.46)	2.11 (1.88-2.37)
Composite of bleeding, venous and arterial thromboembolism										
No. of events	19	14	20	14	14	621	624	597	591	553
IR (95% CI), per 100 PYs	6.06 (3.65-9.46)	4.49 (2.45-7.53)	6.44 (3.93-9.94)	4.53 (2.47-7.60)	4.56 (2.49-7.64)	5.46 (5.04-5.91)	5.53 (5.11-5.99)	5.34 (4.92-5.78)	5.33 (4.91-5.78)	5.03 (4.62-5.47)
30-d cumulative incidence per 1000 (95%CI)	4.95 (3.10-7.62)	3.67 (2.12-6.05)	5.26 (3.34-8.01)	3.70 (2.14-6.10)	3.72 (2.15-6.13)	4.46 (4.12-4.82)	4.52 (4.18-4.88)	4.36 (4.02-4.72)	4.35 (4.01-4.71)	4.11 (3.78-4.46)
All-cause mortality										
No. of events	22	13	21	19	26	1,128	1,139	1,155	1,134	1,117
IR (95% CI), per 100 PYs	7.00 (4.39-10.60)	4.16 (2.21-7.11)	6.75 (4.18-10.31)	6.13 (3.69-9.58)	8.45 (5.52-12.38)	9.90 (9.33-10.50)	10.08 (9.51-10.69)	10.31 (9.72-10.92)	10.21 (9.62-10.82)	10.14 (9.56-10.75)
30-d cumulative incidence, per 1000 (95%CI)	0.57 (0.33-0.81)	0.34 (0.16-0.53)	0.55 (0.32-0.79)	0.50 (0.28-0.73)	0.69 (0.43-0.96)	0.81 (0.76-0.86)	0.82 (0.78-0.87)	0.84 (0.79-0.89)	0.84 (0.79-0.88)	0.83 (0.78-0.88)

IR, incidence rate; PYs, person-years; TIA, transient ischemic attack.

a Cells containing <10 individuals are masked according to Statistics Netherlands’ privacy policy. To prevent recalculation of cells <10, sometimes corresponding cells are additionally masked.

In line with this result, the risk of adverse clinical events remained stable across Ramadan in the native Dutch cohort, with 30-day cumulative incidences ranging from 4.11 to 4.52 per 1000 for the composite of bleeding and venous and arterial thromboembolism, and from 0.81 to 0.84 per 1000 for all-cause mortality (Figure 4B).

### 3.4 Subgroup analysis: Moroccan cohort

The subgroup analysis restricted to VKA users in the Ramadan cohort with a Moroccan background (*n* = 1492) also demonstrated a higher proportion of clinically relevant dose reductions during the Ramadan month (4.3%) than the other months (range, 2.7%-3.6%), as well as a lower proportion of clinically relevant dose increases (1.6% during Ramadan vs 1.9%-3.8% in other months; Supplementary Table 5). In addition, the mean proportion of supratherapeutic INRs was numerically the highest during Ramadan (20.4% ± 33.0%), with a difference of 0.8% (95% CI, −1.6% to 3.2%) compared with the first observation period. A similar pattern was observed for the percentage of time spent above the target range. TTRs in the 5 observation periods were generally low, with a mean of 46.6 ± 34.8 during the Ramadan month. INR variability remained similar across Ramadan, with a mean of 1.3 ± 6.1 during Ramadan.

## 4 Discussion

Our cohort study examined the quality of VKA treatment and clinical outcomes across Ramadan at a population level in the Netherlands. In the Ramadan cohort, no apparent differences were observed in TTR across Ramadan. The percentage of INRs above target range was slightly higher during and after Ramadan than before Ramadan. Notably, a higher percentage of VKA users received a clinically relevant dose reduction during Ramadan than in the consecutive months, while a lower percentage received a relevant dose increase after Ramadan. INR variability remained stable over the 5-month follow-up. We observed similar incidences of clinical outcomes, including all-cause mortality, bleeding, and thromboembolic events, across Ramadan.

As survey data from The Netherlands Institute for Social Research indicated higher participation in Ramadan fasting among Dutch Moroccan Muslims compared to other Muslim groups in the Netherlands [58], we performed a subgroup analysis among VKA users with a Moroccan background, the results of which were similar to our primary analysis. We also studied a negative control native Dutch cohort, and as expected, no apparent changes were observed in either VKA treatment quality or risk of adverse clinical events across Ramadan.

Overall, we observed only minor Ramadan-related changes in the quality of VKA treatment. A possible explanation could be that anticoagulation clinics anticipate possible Ramadan-related changes in vitamin K and tablet intake when monitoring patients who participate in Ramadan fasting. In addition, in this patient group, physicians may be sooner inclined to perform dose adjustments upon a slight INR increase. In line with this hypothesis, we observed that a higher proportion of VKA users received a clinically relevant dose reduction during the month of Ramadan than in consecutive months. In addition, no such time-related pattern was observed across Ramadan in the native Dutch cohort, where the percentage of VKA users who received a clinically relevant dose reduction was also slightly lower compared to the Ramadan cohort.

However, possible changes over time unrelated to Ramadan could have contributed to our observations, such as seasonal variability in VKA stability. For example, respiratory tract infections, with or without antibiotic exposure, and fever are associated with increased risk of supratherapeutic INRs [65,66]. To account for possible time-related factors possibly affecting our study outcomes, we additionally identified a native Dutch cohort. As most time-related changes observed in the Ramadan cohort, such as changes in proportions of relevant dose adjustments and proportions of INRs within or above target range, were not observed in the negative control native Dutch cohort, this argues against a major impact of time-related factors.

Our study contributes to the existing literature on the quality of VKA treatment during Ramadan. A study among Muslim warfarin users who were clinically assessed to be fit to fast (*n* = 30) performed weekly INR measurements during a 3-month study period (ie, before, during, and after Ramadan), the results of which were masked to patients and their physicians [35]. The mean INR increased during Ramadan compared with the month before Ramadan and returned to the pre-Ramadan level thereafter [35]. Changes were also observed in the TTR, which was lower during and after Ramadan compared with the month preceding Ramadan, with a substantial increase in TAR during the Ramadan month and TBR during the post-Ramadan month [35]. This study was not powered to assess possible effects of Ramadan fasting on clinical outcomes. Other studies also reported more frequent supratherapeutic INRs during the Ramadan and post-Ramadan month [41] and an increase in the mean INR and lower TTR during and after Ramadan compared with before Ramadan [40].

Our study is strengthened by its design, comparing the study outcomes before, during, and after Ramadan in the same cohort of VKA users who were likely to fast during Ramadan, thereby limiting bias due to (time-fixed) confounding. The native Dutch cohort served as a negative control, examining possible changes over time unrelated to Ramadan. However, several limitations need to be taken into account when interpreting our study findings. First, we identified a cohort of VKA users who were likely to fast during Ramadan based on the proxy of immigration background combined with country of origin because no individual-level data on religious practice or fasting behavior were available. Specifically, we defined this as being born in (or having parents from) a country where ≥85% of the population identifies as Muslim. We acknowledge that this approach carries a risk of cultural stereotyping by inferring religious practice from family origin.

Moreover, our pragmatic proxy for potential fasting could have led to misclassification in both the Ramadan cohort and the native Dutch cohort, ie, patients in the Ramadan cohort might not have fasted during Ramadan and vice versa. In addition, some VKA users might be exempted from fasting because of their health status and therefore might not have fasted during Ramadan. As this misclassification would primarily dilute the results, the observed Ramadan-related changes would present an underestimation of the true effect. A qualitative study performed among Muslims with diabetes in the Netherlands of Moroccan or Turkish descent explored factors involved in the decision-making process about whether to fast during Ramadan [67]. Many participants indicated that deciding to refrain from fasting is difficult, can lead to feelings of guilt and shame, and that there is uncertainty about whether conditions are severe enough to be exempted from fasting [67]. Furthermore, studies among Muslims with diabetes indicate that fasting against advice from healthcare professionals may occur [68,69]. Although some VKA users might be exempted from fasting, changes in diet, timing of food and oral medication intake, sleep pattern, and physical activity may still occur during Ramadan, especially when surrounded by fasting relatives [67]. In addition, we expect the amount of misclassification in the native Dutch cohort to be minimal, as only a very low proportion of native Dutch inhabitants (estimated at about 0.2%) consider themselves Muslim [58].

Second, clinical events that did not result in a hospital admission and were diagnosed in primary care, such as deep vein thrombosis, TIA, or bleeding events, were missing, which might have led to an underestimation of the true event rates. However, as we observed the cohorts both before, during, and after Ramadan, this misclassification may not have affected comparisons over time.

The increase in the average percentage of supratherapeutic INRs during and after Ramadan we observed is in line with previous studies, possibly attributable to a decrease in dietary vitamin K intake and changes in timing of VKA intake, and might possibly place patients at increased risk of bleeding. Although we did not observe changes in incidences of adverse clinical outcomes across Ramadan, these estimates likely present an underestimation. Nevertheless, clinical guidelines or recommendations specifically addressing VKA management during Ramadan fasting are lacking, whereas these type of guidelines are available for diabetes [70], chronic kidney disease [71], and for broader cardiovascular disease management [33]. A survey conducted in 7 countries reported that patients with diabetes who received Ramadan-specific diabetes education before Ramadan better adhered to Ramadan-specific diabetes management recommendations than patients who did not receive such education, such as altering drug dosage and timing before Ramadan, blood glucose monitoring during fasting, and breaking the fast if symptoms of hypo- or hyperglycemia develop [72].

In conclusion, in a cohort of VKA users who were likely to fast during Ramadan in the Netherlands, both VKA treatment quality and risks of adverse clinical events remained largely unchanged across Ramadan, except for a slightly higher proportion of supratherapeutic INRs during Ramadan together with more frequent dose reductions. These findings could serve to increase awareness for possible Ramadan-related changes in patients treated with VKAs. Healthcare professionals involved may more frequently ask VKA users whether they plan to participate in Ramadan fasting, consider more intensive INR monitoring, or anticipate possible Ramadan-related changes in VKA dosing. Future research should prioritize collecting self-reported fasting status to improve the accuracy of exposure assessment and better capture religious and personal heterogeneity.
