# Supplementary material for: Changes in quality of vitamin K antagonist treatment and clinical outcomes during Ramadan: a Dutch population-based cohort study
Source: Res Pract Thromb Haemost. 2025 Aug 13;9(6):103010. doi: 10.1016/j.rpth.2025.103010 (PMC12451347; doi:10.1016/j.rpth.2025.103010)
Supplement: Supplementary Figures and Tables [file mmc1.pdf]

Table of Content

Supplementary Methods 2
Data sources 2
Dutch anticoagulation clinics 2
Statistics Netherlands (in Dutch “Centraal Bureau voor de Statistiek”, CBS) 2
Identification of Ramadan cohort 3
Supplementary Table 1. Determination of country of origin 3
Supplementary Table 2. Codes for variable identification 4
Supplementary Results 7
Supplementary Table 3. Additional baseline characteristics of individuals receiving vitamin K antagonist treatment in the Ramadan cohort 7
Supplementary Table 4. Baseline characteristics of individuals receiving vitamin K antagonist treatment in the native Dutch cohort 8
Supplementary Table 5. Vitamin K antagonist treatment across Ramadan among VKA users in the Ramadan cohort with a Moroccan background 9
References 11

Supplementary Methods

Data sources

Dutch anticoagulation clinics

The following 17 Dutch anticoagulation clinics participated in the current study: Stichting Trombosedienst Leiden e.o., Trombosedienst St. Anna Ziekenhuis, Trombosedienst Delft, Trombosediensten Winterswijk, Starlet Diagnostisch Centrum, Trombosedienst Unilabs Midden, Trombosedienst Ropcke-Zweers Ziekenhuis, Trombosedienst voor het Gooi, Atalmedial, Trombosedienst Maastricht, Trombosedienst Zuyderland, Trombosedienst Franciscus Gasthuis & Vlietland, Stichting Zeister Trombosedienst, Trombosedienst Isala Zwolle, Trombosedienst N.W.Veluwe, Trombosedienst Diagnost-IQ, STAR-SHL. In the Netherlands, anticoagulation clinics monitor vitamin K antagonist (VKA) treatment of patients who live in well-defined geographical areas. These clinics provided information on VKA treatment, including start- and end dates, indications for VKA treatment, international normalized ratio (INR) measurements with corresponding INR target ranges, and type of VKA used. The clinics are managed by the Dutch Federation of Anticoagulation Clinics (in Dutch "Federatie Nederlandse Trombosediensten", FNT).

Statistics Netherlands (in Dutch "Centraal Bureau voor de Statistiek", CBS)

Personal characteristics

Statistics Netherlands provides data on personal characteristics, which were collected at the start of follow-up (i.e., d_0), such as year of birth, sex, immigration background, country of origin, collected from the Personal Records Database (in Dutch "Basisregistratie Personen", BRP)[1]. This dataset includes all persons who have been registered in the BRP since October 1994, both residents (i.e., individuals who were registered in the population register of a Dutch municipality) and non-residents (i.e., individuals who had a relationship with the Dutch government)[2]. Persons who would stay in the Netherlands for more than four months are compulsory to register at a Dutch municipality and therefore their demographic characteristics would be recorded in the BRP. For the current study, data from the calendar years 2013 to 2019 were used.

Household income

Data on household income were collected from the Tax and Customs administration and student grant registrations of the Education Executive Agency ("Dienst Uitvoering Onderwijs", DUO)[2,3].

Outpatient medication prescriptions

Data on outpatient dispensed medication prescriptions for which costs were reimbursed under the basic health insurance in the Netherlands for the population registered in the Municipal Population Register are collected from the Health Insurance Board[4] (MEDICIJNTAB). In the data, only the year of a dispensed prescription is available. For antithrombotic agents, data on outpatient dispensed prescriptions were identified by the codes from the Anatomic-Therapeutic-Chemical (ATC) system of the World Health Organization (i.e., B01A*). The data source is the same as that of the MEDICIJNTAB dataset, but the data is more granular, in which the date of each dispensed prescription is available, but the amounts of dispensings are unavailable. Medications received in hospitals and in nursing homes are not included, while those received in residential homes for elderly are included[5].

In-hospital diagnoses

Data on diagnoses registered with hospital admissions in Dutch hospitals collected from the National Basic Register of Hospital Care of Dutch Hospital Data which include all general and academic Dutch hospitals and two short-stay categorical hospitals (i.e., a cancer clinic and an eye hospital)[2]. This dataset includes information for persons registered in the BRP and provides data on diagnoses retrieved from discharge letters, length of hospital stay, and date of admission/discharge[6-9].

Mortality and cause of death

Mortality data includes information on the dates of death for all persons who have been registered in the BRP since October 1994[10]. All underlying causes of death of persons who were registered in the BRP and died since the 2013 statistical year were collected from the nationwide Dutch Registry of Causes of Death statistics[11]. In addition, the most important injury and the location of the accident are included for those who died of non-natural death. For all deceased persons, the location of death and the statistical year, or the year in which the deceased is included in the statistics, is also present.

Identification of Ramadan cohort

Statistics Netherlands does not provide data on religion nor participation in Ramadan fasting, therefore, VKA users were classified into the Ramadan cohort based on an immigration background (first- or second-generation) from a country of origin where the Muslim population accounts for ≥85% of the total population. These countries† are (ranked by proportion of Muslim population): Maldives, Mauritania, Somalia, Afghanistan, Iran, Western Sahara, Algeria, Morocco, Comoros, Niger, Tajikistan, Tunisia, Palestine, Azerbaijan, Jordan, Senegal, Yemen, Djibouti, Libya, Mayotte, Pakistan, Gambia, Saudi Arabia, Sudan, Iraq, Mali, Turkmenistan, Kosovo, Bangladesh, Egypt, Guinea, Turkey, Uzbekistan, Indonesia, Oman, and Syria.

†Data source: https://en.wikipedia.org/wiki/Islam_by_country (accessed on 09/08/2024).

Country of origin was determined by the variable GBAHERKOMSTLAND in the CBS dataset GBAPERSOONTAB of statistical year 2023, which refers to the country where a person was born, or where the parents were born if the person was born in the Netherlands.

Supplementary Table 1. Determination of country of origin

A person was born in the Netherlands	The person's mother was born in the Netherlands	Country of origin of the person
No	Not relevant	The country the person was born
Yes	No	The country the person's mother was born
Yes	Yes	The country the person's father was born

In the study, the Native Dutch cohort was identified by Nederland ('6030'), and the Ramadan cohort was identified by Maldives ('Maldiven', 7041), Mauritania ('Mauritanië', 6020), Somalia ('Somalia', 6013), Afghanistan ('Afganistan', 6023), Iran ('Iran', 5012), Western Sahara ('Westelijke Sahara', 9093), Algeria ('Algerije', 6047), Morocco ('Marokko', 5022), Comoros ('Comoren', 5060), Niger ('Niger', 6040), Tajikistan ('Tadzjikistan', 6057), Tunisia ('Tunesië', 5008), Palestine ('Palestina', 7060), Azerbaijan ('Azerbajdsjan', 5097), Jordan ('Jordanië', 6042), Senegal ('Senegal', 7021), Yemen ('Jemen', 5048), Djibouti ('Djibouti', 9087), Libya ('Libië', 6006), Mayotte ('Mayotte', 5084), Pakistan ('Pakistan', 7020), Gambia ('Gambia', 7008), Saudi Arabia ('Saoediarabië', 5018), Sudan ('Soedan', 7034), Iraq ('Irak', 5043), Mali ('Mali', 5029), Turkmenistan ('Toerkmenistan', 6063), Kosovo ('Kosovo', 5105), Bangladesh ('Bangladesh', 7084), Egypt ('Egypte', 7014), Guinea ('Guinee', 7040), Turkey ('Turkije', 6043), Uzbekistan ('Oezbekistan', 6050), Indonesia ('Indonesië', 6024), Oman ('Oman', 7051), and Syria ('Syrië', 7009).

Individuals whose country of origin was imputed (based on the variable GBAIMPUTATIECODE), or was not one of the selected countries for defining the Dutch cohort and the Ramadan cohort were excluded. In addition, individuals whose country of origin was the Netherlands but were not indicated as native Dutch (based on the variable GBAGENERATIE), or individuals whose country of origin was one of the countries for defining the Ramadan cohort but were indicated as native Dutch, were also excluded.

Supplementary Table 2. Codes for variable identification

Variables	Code version	Code(s)
**Oral anticoagulant**		
Vitamin K antagonist	ATC	B01AA
Direct oral anticoagulant	ATC	B01AE (including B01AF)
**Comorbidities/medical history**		
Myocardial infarction (history)	ICD-10	I21, I22, I23, I252
	ICD-9	410, 4110, 412
Ischemic stroke (history)	ICD-10	H341, I63, I64, I693, I694
	ICD-9	3623, 434, 436, 438
TIA	ICD-10	G45
	ICD-9	435
Venous thromboembolism	ICD-10	G08, G951, I26, I636, I676, I801, I802, I803, I808, I809, I81, I820, I821, I822, I823, I828, I829, K550
	ICD-9	325, 3361, 4151, 4340, 4376, 4511, 4512, 4518, 4519, 452, 4530, 4531, 4532, 4533, 4534, 4535, 4537, 4538, 4539, 5570
Systemic thromboembolism	ICD-10	I74
	ICD-9	444
Peripheral artery disease	ICD-10	I739
	ICD-9	4439
Major bleeding	ICD-10	D62, D683, H313, H356, H431, H450, H922, I312, I60, I61, I62, I850, I9820, I983, J942, K2210, K2212, K2214, K2216, K228, K250, K252, K254, K256, K260, K262, K264, K266, K270, K272, K274, K276, K280, K282, K284, K286, K290, K3180, K5520, K625, K6380, K661, K920, K921, K922, M250, N02, N938, N939, N950, R04, R31, R58, S064, S065, S066
	ICD-9	2851, 28659, 2878, 2879, 36281, 3636, 37923, 38869, 4230, 430, 431, 4320, 4321, 4329, 4590, 53082, 5310, 5312, 5314, 5316, 5320, 5322, 5324, 5326, 5330, 5332, 5334, 5336, 5340, 5342, 5344, 5346, 5693, 5780, 5781, 5789, 5967, 5997, 6271, 7191, 7848, 7863, 852, 853, 8602, 8603, 8604, 8605
Gastrointestinal bleeding	ICD-10	I850, I9820, I983, K2210, K2212, K2214, K2216, K228, K250, K252, K254, K256, K260, K262, K264, K266, K270, K272, K274, K276, K280, K282, K284, K286, K290, K3180, K5520, K625, K6380, K920, K921, K922
	ICD-9	53082, 5310, 5312, 5314, 5316, 5320, 5322, 5324, 5326, 5330, 5332, 5334, 5336, 5340, 5342, 5344, 5346, 5693, 5780, 5781, 5789
Intracranial hemorrhage	ICD-10	I60, I61, I62, S064, S065, S066
	ICD-9	430, 431, 4320, 4321, 4329, 852, 853
Atrial fibrillation	ICD-10	I48
	ICD-9	4273
Heart failure	ICD-10	I50
	ICD-9	428
Rheumatic mitral stenosis/mechanical heart valves	ICD-10	I050, I052, I059, Z952
	ICD-9	3940, 3942, 3949, V433
Other valvular heart diseases	ICD-10	I051, I058, I06, I07, I08, I34, I35, I36, I37, I38, I39
	ICD-9	3941, 395, 396, 397, 424
Liver diseases	ICD-10	B15, B16, B17, B18, B19, C22, I85, I982, I983, K7, Z944
	ICD-9	070, 155, 4560, 4561, 4562, 571, 572, 573, 7824, V427

Kidney diseases	ICD-10	E102, E112, E122, E132, E142, I120, I13, N0, N1, N26, N27, Q60, Q61, T824, T861, Y602, Y612, Y622, Y841, Z49, Z940, Z992
	ICD-9	403, 404, 58, V420, V451, V56
Anemia	ICD-10	D5, D60, D61, D62, D63, D64
	ICD-9	280, 281, 282, 283, 284, 285
Diabetes	ICD-10	E10, E11, E12, E13, E14
	ICD-9	250
Chronic obstructive pulmonary disease	ICD-10	J44
	ICD-9	496
Thyroid disease	ICD-10	E0
	ICD-9	240, 241, 242, 243, 244, 245, 246
Parkinson’s disease	ICD-10	G20
	ICD-9	3320
Alzheimer’s disease	ICD-10	G30
	ICD-9	3310
Autoimmune diseases	ICD-10	D8
	ICD-9	279
Malignant tumor	ICD-10	C
	ICD-9	14, 15, 16, 17, 18, 19, 20
**CHA_2DS_2-VASc score**		
Heart failure	ICD-10	I50
	ICD-9	428
Hypertension	ICD-10	I10, I11, I12, I13, I15
	ICD-9	401, 402, 403, 404, 405
Diabetes	ICD-10	E10, E11, E12, E13, E14
	ICD-9	250
Stroke/TIA/thromboembolism	ICD-10	G45, H341, I63, I64, I693, I694, I74
	ICD-9	3623, 434, 435, 436, 438, 444
Vascular disease	ICD-10	I21, I22, I23, I250, I251, I252, I70, I739
	ICD-9	410, 4110, 412, 4140, 4143, 4144, 440, 4439
**HAS-BLED score**		
Hypertension (uncontrolled)	ATC	C02, C03, C07, C08, C09<br>(at least 3 classes of antihypertensive drugs)
Kidney diseases	ICD-10	E102, E112, E122, E132, E142, I120, I13, N0, N1, N26, N27, Q60, Q61, T824, T861, Y602, Y612, Y622, Y841, Z49, Z940, Z992
	ICD-9	58, 403, 404, V420, V451, V56
Liver diseases	ICD-10	B15, B16, B17, B18, B19, C22, K7, I85, I982, I983, Z944
	ICD-9	070, 155, 4560, 4561, 4562, 571, 572, 573, 7824, V427
Ischemic stroke/TIA	ICD-10	I63, I64, H341, I693, I694, G45
	ICD-9	434, 436, 3623, 438, 435
Major Bleeding	ICD-10	D62, D683, H313, H356, H431, H450, H922, I312, I60, I61, I62, I850, I9820, I983, J942, K2210, K2212, K2214, K2216, K228, K250, K252, K254, K256, K260, K262, K264, K266, K270, K272, K274, K276, K280, K282, K284, K286, K290, K3180, K5520, K625, K6380, K661, K920, K921, K922, M250, N02, N938, N939, N950, R04, R31, R58, S064, S065, S066
	ICD-9	430, 431, 852, 853, 2851, 2878, 2879, 3636, 4230, 4320, 4321, 4329, 4590, 5310, 5312, 5314, 5316, 5320, 5322, 5324, 5326, 5330, 5332, 5334, 5336, 5340, 5342, 5344, 5346, 5693, 5780, 5781, 5789, 5967, 5997, 6271, 7191, 7848, 7863, 8602, 8603, 8604, 8605, 28659, 36281, 37923, 38869, 53082

Alcohol abuse	ICD-10	F10, G312, G621, G721, I426, K292, K70, K860, K852, Q860, P043, E244, E52, O354, T51, Z714, Z721
	ICD-9	291, 303, 3050, 3575, 4255, 5353, 5710, 5711, 5712, 5713, 6554, 76071, 2652, 980
Antiplatelet drug or NSAIDs	ATC	B01AC, M01A
**Other antithrombotic agent**		
Heparin group	ATC	B01AB
Antiplatelet drug	ATC	B01AC
**Clinical event**		
Ischemic stroke/TIA/ thromboembolism/Myocardial infarction	ICD-10	I63, I64, H341, G45, I74, I21, I22
Venous thromboembolism	ICD-10	G08, G951, I26, I636, I676, I801, I802, I803, I808, I809, I81, I820, I821, I822, I823, I828, I829, K550
Major bleeding	ICD-10	D62, D683, H313, H356, H431, H450, H922, I312, I60, I61, I62, I850, I9820, I983, J942, K2210, K2212, K2214, K2216, K228, K250, K252, K254, K256, K260, K262, K264, K266, K270, K272, K274, K276, K280, K282, K284, K286, K290, K3180, K5520, K625, K6380, K661, K920, K921, K922, M250, N02, N938, N939, N950, R04, R31, R58, S064, S065, S066

Abbreviations: ATC, Anatomical Therapeutic Chemical Classification System; ICD, International Classification of Diseases; TIA, transient ischemic attack; NSAID, nonsteroidal anti-inflammatory drug.

Supplementary Results

Supplementary Table 3. Additional baseline characteristics of individuals receiving vitamin K antagonist treatment in the Ramadan cohort

**Cohort**	**Ramadan cohort (N=3,835)**
**Comorbidity[a], N (%)**	
≥1 comorbidity[b]	2259 (58.9)
History of myocardial infarction	497 (13.0)
History of ischemic stroke	110 (2.9)
History of TIA	18 (0.5)
History of ischemic stroke/TIA	125 (3.3)
Venous thromboembolism	225 (5.9)
Systemic thromboembolism	53 (1.4)
Peripheral artery disease	49 (1.3)
Major bleeding	238 (6.2)
Gastrointestinal bleeding	93 (2.4)
Intracranial haemorrhage	28 (0.7)
Atrial fibrillation	1116 (29.1)
Heart failure	668 (17.4)
Rheumatic mitral stenosis/mechanical heart valves	276 (7.2)
Other valvular heart diseases	433 (11.3)
Liver diseases	115 (3.0)
Kidney diseases	477 (12.4)
Anemia	494 (12.9)
Diabetes	840 (21.9)
Chronic obstructive pulmonary disease	231 (6.0)
Thyroid disease	81 (2.1)
Parkinson’s disease	17 (0.4)
Alzheimer’s disease	<10[d]
Autoimmune diseases	22 (0.6)
Malignant tumor	137 (3.6)
**Prescribed antithrombotic agent within 6 months before d_0[c], N (%)**	
Low-molecular-weight heparin (≥1 prescription)	396 (10.3)
Antiplatelet agents (≥1 prescription)	524 (13.7)
**Type of most recent prescribed anticoagulant before d_0[c], N (%)**	
VKA only	3730 (97.3)
VKA + Low-molecular-weight heparin	34 (0.9)
Low-molecular-weight heparin only	71 (1.9)

[a] Comorbidities were identified by in-hospital diagnoses within 3 years before the start of follow-up (d_0).
[b] Including Myocardial infarction (history), Ischemic stroke/Transient ischemic attack, Venous thromboembolism, Systemic thromboembolism, Peripheral artery disease, Major bleeding, Atrial fibrillation, Heart failure, Rheumatic mitral stenosis/mechanical heart valves, Other valvular heart diseases, Liver diseases, Kidney diseases, Anemia, Diabetes, Chronic obstructive pulmonary disease, Thyroid disease, Parkinson's disease, Alzheimer's disease, Autoimmune diseases, and Malignant tumor.
[c] The date d_0 refers to the date 60 days before the start of Ramadan in a particular calendar year.
[d] Cells containing <10 individuals are masked according to Statistics Netherlands’ privacy policy.
Abbreviations: VKA = vitamin K antagonist; TIA = transient ischemic attack.

Supplementary Table 4. Baseline characteristics of individuals receiving vitamin K antagonist treatment in the native Dutch cohort

Cohort	Native Dutch cohort (N=139,207)
**Comorbidity[a], N (%)**	
≥1 comorbidity[b]	71562 (51.4)
History of myocardial infarction	12174 (8.7)
History of ischemic stroke	4917 (3.5)
History of TIA	1904 (1.4)
History of ischemic stroke/TIA	6558 (4.7)
Venous thromboembolism	6028 (4.3)
Systemic thromboembolism	2174 (1.6)
Peripheral artery disease	2586 (1.9)
Major bleeding	6968 (5.0)
Gastrointestinal bleeding	2822 (2.0)
Intracranial haemorrhage	774 (0.6)
Atrial fibrillation	39966 (28.7)
Heart failure	15571 (11.2)
Rheumatic mitral stenosis/mechanical heart valves	4063 (2.9)
Other valvular heart diseases	10653 (7.7)
Liver diseases	1751 (1.3)
Kidney diseases	12703 (9.1)
Anemia	9196 (6.6)
Diabetes	14174 (10.2)
Chronic obstructive pulmonary disease	9048 (6.5)
Thyroid disease	2309 (1.7)
Parkinson's disease	604 (0.4)
Alzheimer's disease	520 (0.4)
Autoimmune diseases	258 (0.2)
Malignant tumor	9333 (6.7)
**Prescribed antithrombotic agent within 6 months before d_0[c], N (%)**	
Low-molecular-weight heparin (≥1 prescription)	11506 (8.3)
Antiplatelet agents (≥1 prescription)	12786 (9.2)
**Type of most recent prescribed anticoagulant before d_0[c], N (%)**	
VKA only	135501 (97.3)
VKA + Low-molecular-weight heparin	1025 (0.7)
Low-molecular-weight heparin only	2681 (1.9)

[a] Comorbidities were identified by in-hospital diagnoses within 3 years before the start of follow-up (d_0).
[b] Including Myocardial infarction (history), Ischemic stroke/Transient ischemic attack, Venous thromboembolism, Systemic thromboembolism, Peripheral artery disease, Major bleeding, Atrial fibrillation, Heart failure, Rheumatic mitral stenosis/mechanical heart valves, Other valvular heart diseases, Liver diseases, Kidney diseases, Anemia, Diabetes, Chronic obstructive pulmonary disease, Thyroid disease, Parkinson's disease, Alzheimer's disease, Autoimmune diseases, and Malignant tumor.
[c] The date d_0 refers to the date 60 days before the start of Ramadan in a particular calendar year.
Abbreviations: VKA = vitamin K antagonist; TIA = transient ischemic attack.

Supplementary Table 5. Vitamin K antagonist treatment across Ramadan among VKA users in the Ramadan cohort with a Moroccan background

Cohort	Ramadan cohort (subgroup Moroccan)				
**Observation period**	**-2 month**	**-1 month**	**Ramadan**	**+1 month**	**+2 month**
**Number of INR measurements**	1492	1448	1418	1378	1353
Median (IQR)	2.0 (1.0-3.0)	1.0 (1.0-2.0)	2.0 (1.0-2.0)	1.0 (1.0-2.0)	1.0 (1.0-2.0)
**Mean interval between INR measurement (days)**	1333	1204	1156	1083	1007
Median (IQR)	17.0 (10.5-25.5)	16.3 (11.0-22.0)	16.3 (10.7-22.1)	16.0 (11.0-23.0)	17.5 (11.7-24.5)
**Median INR value**	1336	1270	1208	1137	1074
Median (IQR)	2.60 (2.20-3.20)	2.60 (2.20-3.15)	2.65 (2.20-3.20)	2.60 (2.20-3.20)	2.60 (2.20-3.10)
Mean±SD	2.74±0.83	2.72±0.73	2.75±0.76	2.73±0.78	2.67±0.73
Fixed effect β (95%CI)	0 (Reference)	-0.021 (-0.073 to 0.031)	0.008 (-0.045 to 0.061)	-0.005 (-0.059 to 0.049)	-0.066 (-0.121 to -0.011)
**Median recommended average dose[a]**	1336	1268	1207	1136	1071
Median (IQR)	2.15 (1.43-3.14)	2.17 (1.43-3.07)	2.12 (1.38-3.00)	2.12 (1.38-3.04)	2.09 (1.39-3.05)
Mean±SD	2.39±1.36	2.38±1.34	2.32±1.30	2.32±1.29	2.30±1.28
Fixed effect β (95%CI)	0 (Reference)	-0.009 (-0.027 to 0.010)	-0.032 (-0.050 to -0.013)	-0.051 (-0.070 to -0.032)	-0.050 (-0.069 to -0.030)
**≥1 clinically relevant dose increase[b]**	1332	1202	1154	1081	1003
N (%)	51 (3.8)	32 (2.7)	18 (1.6)	21 (1.9)	21 (2.1)
**≥1 clinically relevant dose reduction[b]**	1332	1202	1154	1081	1003
N (%)	48 (3.6)	41 (3.4)	50 (4.3)	37 (3.4)	27 (2.7)
**INR variability (VGR)**	1333	1204	1156	1083	1007
Mean±SD	1.2±5.1	1.2±5.5	1.3±6.1	1.2±4.9	0.8±2.3
Fixed effect β (95%CI)	0 (Reference)	-0.027 (-0.380 to 0.326)	0.036 (-0.323 to 0.395)	-0.006 (-0.372 to 0.360)	-0.369 (-0.743 to 0.006)
**Proportion of INRs within target range, %**	1336	1270	1208	1137	1074
Mean±SD	49.9±41.0	52.0±42.4	50.0±41.1	52.4±41.7	52.9±42.8
Fixed effect β (95%CI)	0 (Reference)	2.044 (-0.958 to 5.046)	-0.074 (-3.119 to 2.971)	2.471 (-0.629 to 5.571)	2.864 (-0.288 to 6.016)
**Proportion of INRs below target range, %**	1336	1270	1208	1137	1074
Mean±SD	30.5±37.7	29.0±38.2	29.6±37.5	27.8±37.6	30.0±38.6
Fixed effect β (95%CI)	0 (Reference)	-1.415 (-4.029 to 1.199)	-0.685 (-3.338 to 1.968)	-2.607 (-5.309 to 0.096)	-0.338 (-3.087 to 2.411)
**Proportion of INRs above target range, %**	1336	1270	1208	1137	1074
Mean±SD	19.6±32.1	19.1±32.6	20.4±33.0	19.8±32.1	17.2±31.1

Fixed effect β (95%CI)	0 (Reference)	-0.585 (-2.952 to 1.781)	0.823 (-1.577 to 3.222)	0.171 (-2.271 to 2.612)	-2.467 (-4.949 to 0.015)
**Proportion of INRs ≥5, %**	1336	1270	1208	1137	1074
Mean±SD	3.0±12.7	2.4±10.7	3.0±12.3	2.9±12.0	2.0±10.4
**Proportion of INRs ≥8, %**	1336	1270	1208	1137	1074
Mean±SD	0.3±4.0	0.1±2.2	0.2±2.6	0.2±3.5	0.1±1.3
**Time in target range, %**	1326	1201	1150	1075	1000
Mean±SD	46.5±35.0	47.7±35.2	46.6±34.8	48.5±35.1	49.4±36.3
Fixed effect β (95%CI)	0 (Reference)	1.316 (-1.236 to 3.869)	0.316 (-2.277 to 2.910)	2.164 (-0.481 to 4.810)	3.194 (0.491 to 5.897)
**Time below target range, %**	1326	1201	1150	1075	1000
Mean±SD	32.1±35.8	30.3±34.9	30.1±34.7	28.9±33.8	30.5±35.2
Fixed effect β (95%CI)	0 (Reference)	-1.828 (-4.283 to 0.628)	-2.092 (-4.591 to 0.406)	-3.268 (-5.816 to -0.719)	-1.633 (-4.238 to 0.972)
**Time above target range, %**	1326	1201	1150	1075	1000
Mean±SD	21.5±30.7	21.6±30.9	22.7±31.4	21.8±30.2	18.9±29.4
Fixed effect β (95%CI)	0 (Reference)	0.072 (-2.188 to 2.332)	1.137 (-1.157 to 3.431)	0.281 (-2.058 to 2.620)	-2.647 (-5.036 to -0.257)
**Changed VKA type**	1332	1200	1152	1081	1002
N (%)	<10[c]	<10[c]	<10[c]	<10[c]	<10[c]
**Switch to DOACs**	1492	1448	1418	1378	1353
N (%)	<10[c]	<10[c]	<10[c]	<10[c]	<10[c]

[a] Recommended average dose is expressed as number of tablets, where one tablet acenocoumarol contains 1 mg and one tablet phenprocoumon 3 mg.

[b] A clinically relevant dose increase or reduction was defined as a change of ≥10% in the average daily VKA dose prescribed at two consecutive INR measurements.

[c] Cells containing <10 individuals were masked according to Statistics Netherlands' privacy policy.

Abbreviations: INR = international normalized ratio; IQR = interquartile range; VGR = variance growth rate; SD = standard deviation; VKA = vitamin K antagonist; DOAC = direct oral anticoagulant.

References

1. Centraal Bureau voor Statistiek. Persoonskenmerken van alle in de Gemeentelijke Basis Administratie (GBA) ingeschreven personen, gecoördineerd. In: Centraal Bureau voor S, (ed). V1 ed: ODISSEI Portal; 2021.
2. Chen Q, van Rein N, van der Hulle T*, et al.* Coexisting atrial fibrillation and cancer: time trends and associations with mortality in a nationwide Dutch study. *Eur Heart J* 2024;**45**:2201-2213. doi:
3. Centraal Bureau voor Statistiek. Inkomen van huishoudens (revisie 2017). In: Centraal Bureau voor Statistiek, (ed). V1 ed: ODISSEI Portal; 2011.
4. Centraal Bureau voor Statistiek. Verstrekkingen van geneesmiddelen op 4 posities ATC-code aan personen. In: Centraal Bureau voor S, (ed). V1 ed: ODISSEI Portal; 2020.
5. Chen Q, Toorop MMA, Tops LF, Lijfering WM, Cannegieter SC. Time Trends in Patient Characteristics, Anticoagulation Treatment, and Prognosis of Incident Nonvalvular Atrial Fibrillation in the Netherlands. *JAMA Netw Open* 2023;**6**:e239973. doi:
6. Centraal Bureau voor Statistiek. Diagnosen behorend bij ziekenhuisopnamen voor RA-gebruik. In: Centraal Bureau voor S, (ed). V1 ed: ODISSEI Portal; 2012.
7. Centraal Bureau voor Statistiek. Diagnosen behorend bij ziekenhuisopnamen Landelijke Basisregistratie Ziekenhuiszorg. In: Centraal Bureau voor S, (ed). V1 ed: ODISSEI Portal; 2019.
8. Centraal Bureau voor Statistiek. Ziekenhuisopnamen voor RA-gebruik. In: Centraal Bureau voor S, (ed). V1 ed: ODISSEI Portal; 2012.
9. Centraal Bureau voor Statistiek. Ziekenhuisopnamen Landelijke Basisregistratie Ziekenhuiszorg. In: Centraal Bureau voor S, (ed). V1 ed: ODISSEI Portal; 2019.
10. Centraal Bureau voor Statistiek. Datum van overlijden van personen die ingeschreven staan in de Gemeentelijke Basisadministratie (GBA). In: Centraal Bureau voor S, (ed). V1 ed: ODISSEI Portal; 2018.
11. Centraal Bureau voor Statistiek. Doodsoorzaken van personen die bij overlijden inwoners waren van Nederland. In: Centraal Bureau voor S, (ed). V1 ed: ODISSEI Portal; 2013.
